# Cryotherapy for the Treatment of Tracheal Stenosis: A Systematic Review

**DOI:** 10.7759/cureus.41012

**Published:** 2023-06-26

**Authors:** Asma Hosna, Muhammad Haseeb ul Rasool, Nicole C Noff, Karim Makhoul, Daniel Miller, Zaryab Umar, Muhammad Ghallab, Rockyb Hasan, Salman Ashfaq, Avish Parikh, Ricardo Lopez

**Affiliations:** 1 Internal Medicine, Icahn School of Medicine at Mount Sinai, New York City, USA; 2 Medicine, Icahn School of Medicine at Mount Sinai, Queens Hospital Center, New York City, USA; 3 Internal Medicine, Icahn School of Medicine at Mount Sinai, Queens Hospital Center, New York City, USA; 4 Neurology, Mount Sinai Queens, New York City, USA; 5 Internal Medicine, Icahn School of Medicine at Mount Sinai, New York City Health and Hospitals, New York City, USA; 6 Internal Medicine, Texas Tech University Health Sciences Center - Amarillo Campus, Lubbock, USA; 7 Internal Medicine, Allama Iqbal Medical College, Lahore, PAK

**Keywords:** radiotherapy, advanced disease, stent placement, lung cancer, cryotherapy

## Abstract

Tracheal stenosis (TS) is an iatrogenic sequela after intubation or tracheostomy that is increasing despite technological improvement and skilled respiratory care in the ICU. According to the studies, the rate of TS varies from 10 to 22%, but only 1-2% of these stenoses are severe and present with inspiratory dyspnea that does not respond to medical management. Bronchoscopy is considered the most appropriate diagnostic test, and laser surgery and tracheobronchial stenting are the most commonly performed procedures for tracheal stenosis. However, alternative treatment options, including cryotherapy for inoperable patients, have yet to be studied widely. As the number of patients requiring ICU admission with mechanical intubation is increasing, it is crucial to acknowledge this complication and consider alternative management options. Here we present a review of the use of cryotherapy for post-intubation tracheal stenosis.

Pubmed, Cochrane, and EMBASE databases were inquired for studies performed using the keywords 'airway stricture' OR 'airway obstruction' AND 'post-intubation' OR 'post-extubation' OR 'tracheostomy' AND 'cryotherapy'. After the primary and secondary screening, five studies were included in the analysis.

We included 67 patients were included in the analysis, with a mean age of 50.2 (range: 42-55) years. Tracheal stenosis and subglottic stricture were the most common sites of stenosis. Twenty-nine patients were treated with cryotherapy only, while the rest 38 patients had cryotherapy followed by balloon dilation. After the intervention, 48 patients experienced improvement, five experienced no change in the symptoms, 13 patients were asymptomatic before the treatment, and one died. No complication was reported in 65 patients, with only minor complications reported in rest.

Although, there is no clear treatment protocol for patients with inoperable tracheal stenosis. Our review demonstrates that cryotherapy for inoperable tracheal stenosis can be an acceptable alternative treatment with significant clinical improvement. Additionally, cryotherapy has fewer adverse effects compared to other treatment options.

## Introduction and background

Prolonged endotracheal intubation is considered one of the most common causes of tracheal stenosis (TS). Tracheal mucosal capillary perfusion pressure varies from 20 to 30 mmHg. When the endotracheal tube's (ETT) cuff pressure exceeds 30 mmHg, it can cause mucosal damage. Short-term ischemia of tracheal epithelium heals with epithelial regeneration, but prolonged ischemia causes damage to the entire thickness of epithelium and submucosa. Long-standing ischemia can lead to ulceration and ischemia of tracheal cartilage, followed by healing by fibrosis, which manifests with varying degrees of circumferential scarring, leading to progressive tracheal stenosis [1]. 

If the cuff pressure is more than 30 mmHg, a longer duration of cuff compression can also lead to ischemic injury to the tracheal rings as nourishment from the mucosa by diffusion is lost. Cartilage damage due to an ischemic injury can be partial or complete thickness. Tracheomalacia, stenosis, and sometimes tracheoesophageal fistula can develop in the affected segments depending on the severity and degree of inflammation [2].

A prospective study by Stauffer et al. demonstrated that in intubation with high-volume, low-pressure cuffed tubes, almost 10-11% of critically ill patients developed tracheal stenosis at the cuff site [3]. Factors responsible for stenosis are cuff pressure, size of the tube compared to tracheal diameter, length of intubation, patient's cardiovascular status, sex and age of the patient, use of high-dose corticosteroid, and development of tracheal mucosal hypoxia [4, 5]. However, tracheal stenosis has been reported in intervals as short as 24 hours post-intubation [6 ].

TS is clinically divided into simple and complex types. Simple TS is less than 1 cm in length ring-shaped circumferential stenosis, and does not involve the cartilage. On the other hand, complex TS is stenosis that involves more than 1 cm in height and is accompanied by cartilage involvement which can present in the form of fibrosis or malacia [2, 7].

During cryotherapy, a cryoprobe is inserted through the working channel of a bronchoscope, and a freeze-thaw effect on the target tissue is created, which facilitates coagulation necrosis and ultimately results in the destruction of the selected area. Available cryogens consist of nitrous oxide, carbon dioxide, and liquid nitrogen. Two methods of tissue removal in cryotherapy exist. The first method induces tissue necrosis by repeated freezing and thawing. The second method is known as a pulling-out method, where frozen tissue is extracted while being attached to the cryoprobe. The latter has a higher risk of bleeding. However, the two methods overall have a low risk of bleeding as the vessels in frozen tissue are vaso-constricted. The cold temperature produces a hemostatic, analgesic, and anti-inflammatory effect. Cryotherapy selectively damages tissue; as a result, it is less likely to damage cartilage, collagen, and fat tissue in the airway. In their recent studies, Browning et al. have shown this device's safety in managing tracheal strictures. Another survey by Finley et al. did not experience any of the complications [8]. This review study reports the success rate and difficulties of using cryotherapy in patients with post-intubation tracheal stenosis. 

## Review

Research question

This systematic review assessed cryotherapy's effectiveness in patients with post-intubation tracheal stenosis. 

Methods

Pubmed, Cochrane Library and EMBASE databases were screened from the inception of the idea through December 4, 2022, using the following keywords: 'airway stricture' OR 'airway obstruction' AND 'post-intubation' OR 'post-extubation' OR 'tracheostomy' AND 'cryotherapy'. The inclusion criteria were as follows: primary studies, case reports or case series, review articles, systemic reviews, meta-analysis, confirmed cases of post-intubation tracheal stenosis, and articles written in English.

The exclusion criteria included studies that were performed in the pediatric population, non-English articles, non-peer-reviewed articles, and guidelines. The articles retrieved were screened by two reviewers, AH and DM, who worked independently to decrease personal review bias. The initial search yielded 116 studies. After the primary screening of the titles and removing duplicates, we screened 94 articles for which the abstract was screened for relevance. Secondary screening yielded 44 articles, for which full-length manuscripts were available, of which five manuscripts were related to the research question. The references of included articles were also screened to eliminate bias and ensure no relevant article was left.

**Figure 1:**
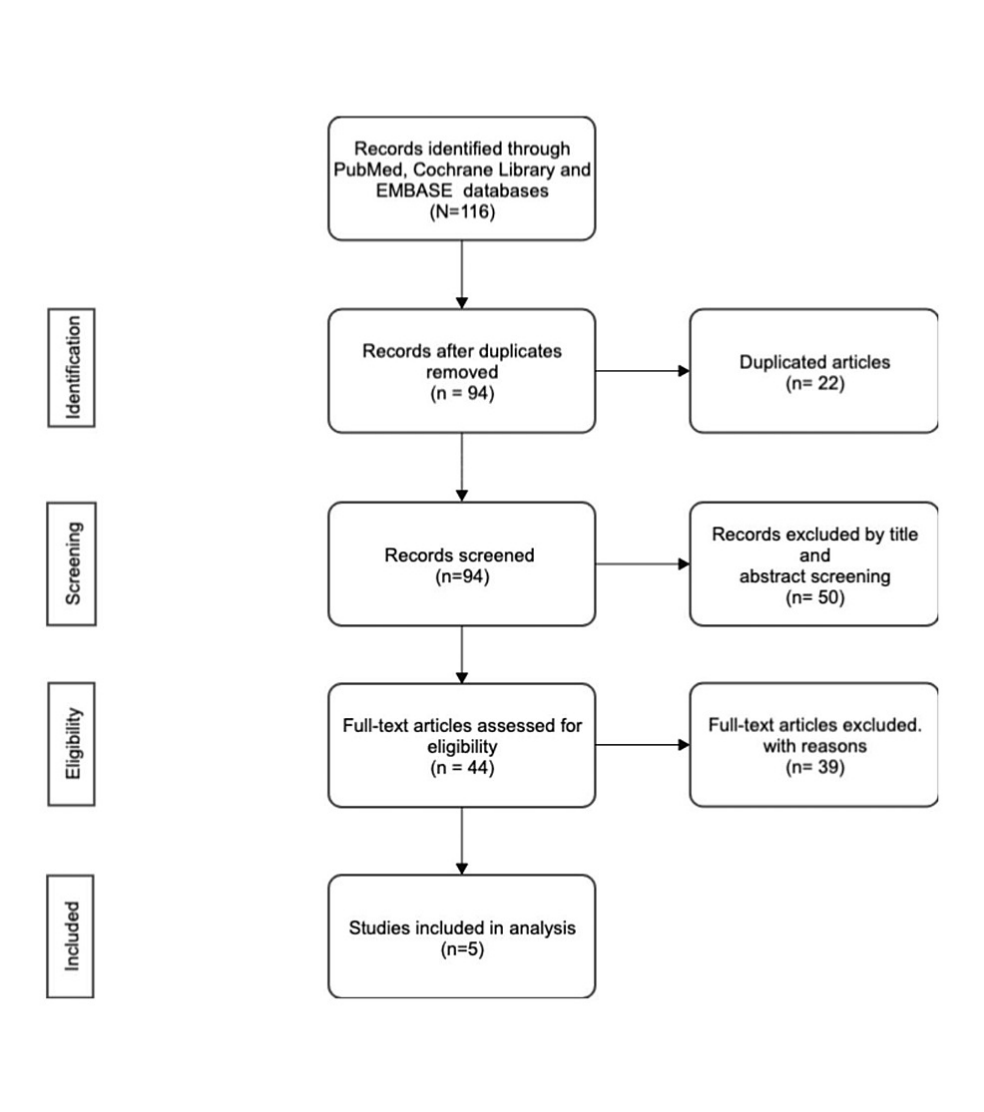
Preferred Reporting Items for Systematic Review and Meta-Analysis (PRISMA) flow diagram of the study screening process

Figure 1 depicts the Preferred Reporting Items for Systematic Review and Meta-analysis (PRISMA) flow diagram of the study screening process.

Result

We included 67 patients in the analysis, with a mean age of 50.2 (42-55) years. Tracheal stenosis and subglottic stricture were the most common sites of stenosis. Twenty-nine patients were treated with cryotherapy only, while the other 38 patients had cryotherapy followed by balloon dilation. After the intervention, 48 patients experienced improvement, 13 patients became asymptomatic, and five experienced no change in the symptoms. One patient died from a different non-related malignancy. No complication was reported in 65 patients, with only minor complications reported in rest. Table 1 summarizes the patient's baseline characteristics, the treatment used, the outcome, and the difficulties of the procedure. 

**Table 1:** Summarizes the patient's baseline characteristics, the treatment used, the outcome, and the complications of the procedure

Author	Age	Number of patients	Most common location of stenosis	Treatment modalities used	Outcome	Complications	
Fernando et al. (2011) [9]	Mean age 51 (18-81) years	35 patients	Subglottic (n=18), tracheal (n=9), and bronchial (n=8)	Spray cryotherapy followed by balloon dilation	13 were asymptomatic, 17 were improved, 4(12.1%) had no improvement or were worse, and 1/33 (3%) patients died	Glottic edema in 1 patient, pneumothorax in 1 patient	
Liu et al. (2015) [10]	49 years, and 62 years	2 patients	Stricture at thyroid isthmus (n=2)	Cryotherapy	Both patients had improved significantly	None	
Jung et al. (2016) [11]	42 years	1 patient	Tracheal	Cryotherapy	Stenosis resolved	None	
Krimsky et al. (2010)[12]	42-year-old female, 74-year-old female, 33-year-old female	3 patients	Subglottic strictures (N=1), glottic strictures (n=1) and vocal cord stenosis (n=1)	Spray cryotherapy alone or in combination with balloon dilation.	Patency of the stenosed areas was achieved	Minimal bleeding	
Bhora et al. (2016)[8]	53 (16-83) years	26 patients	Tracheal stenosis	Spray cryotherapy	88% patients had stenosis at the beginning of the treatment, 4% had stenosis after treatment	None	

Discussion

Laryngotracheal stenosis can be a sequela of either congenital diseases, such as subglottic membranous or cartilaginous narrowing, or acquired pathologies, such as trauma due to prolonged endotracheal intubation, radiation therapy causing tracheal strictures, infection, inflammation, gastroesophageal reflux disease, and inhalational injury [13]. Post-intubation tracheal stenosis occurs mainly from loss of regional blood flow around the cuff site. This causes ischemic injury, and as the damaged tissue heals, it can be associated with the development of fibrosis within 3 to 6 weeks. Risk factors for this development include prolonged duration of intubation, previous history of intubation, increased secretion, complicated infection, hypotension, old age, excessive corticosteroid use, female sex, uncontrolled diabetes mellitus, severe respiratory failure, autoimmune disease, obstructive sleep apnea (OSA), exposure to radiation therapy for oropharyngeal or laryngeal cancer [11]. White et al. reported that tracheal intubation lasting longer than six days increases the risk of stenosis to 5% from 2% for intubation lasting between three to six days. Another study performed by Jung et al. revealed that intubation for as little as 18 hours can lead to the development of tracheal stenosis [12]. 

The patient may not experience any symptoms until the narrowing of the trachea is severe enough to cause symptoms. Almost half of the patients experience dyspnea during exercise, while others experience dyspnea and stridor at rest [13, 14]. Sometimes early symptoms, such as dyspnea during exercise and cough, are confused with diseases such as asthma and COPD or early post-infectious states. Among all the causes of tracheal stenosis, post-intubation tracheal stenosis (PITS) is somewhat avoidable. It can be avoided by selecting the appropriate endotracheal tube size for each patient and close monitoring of the cuff pressure [15]. It takes four to six weeks to develop PITS. A study conducted by Shin et al. on 117 patients with PITS showed that the mean time from tracheal trauma to the diagnosis of PITS was 1.8 months [16]. Similarly, another study by Beyoglu et al. reported that the time to diagnosis was 42.0 ± 1.15 days [2]. 

Although the standard treatment for complex tracheal stenosis is tracheal resection of the stenosed part with end-to-end anastomosis, this is only sometimes feasible [9, 16]. Multiple different options are available for the treatment of tracheal stenosis involving endoscopic procedures, including laser ablation, electrocautery, argon-laser coagulation, application of mitomycin C topically, mechanical approaches including rigid bronchoscopy or balloon dilatation, cold therapies (cryotherapy, cryo-debridement, cryospray), and airway stenting [12, 16, 17, 18]. However, mechanical endoscopic therapies can be problematic and may require repeated interventions, complicating the treatment, and the prognosis becomes poor [19]. The recurrence rate after bronchoscopic dilatation can be as high as 90% in complex TS. Therefore, bronchoscopic balloons or mechanical dilatation should only be considered as a bridge to surgical treatment in severely symptomatic patients [9]. Cryotherapy is a new approach that helps to accelerate the healing response resulting in less fibrosis as the integrity of the extracellular matrix remains largely intact, and an intact stroma provides the structural support for the appropriate wound healing resulting in a decrease in the need or the duration of time to intervention [20]. Rodgers et al. reported the first successful use of endotracheal cryotherapy for the treatment of tracheal stenosis in 1977. In a follow-up study by them, it was proved that cryotherapy was successful in curing airway strictures in 20 out of 24 lesions treated [9].

Initially, cryotherapy was carried out using contact probes. Advances have been made, and spray cryotherapy (SCT) was invented, in which liquid nitrogen is delivered by using a 7F disposable catheter, causing tissue ablation. This modality has outstanding long-term outcomes when used for gastrointestinal procedures; therefore, some centers started using it for airway stricture management, likely to damage cartilage, collagen, and fat tissue in the airway as it destroys tissue selectively [20]. Airway stenting complications include migration, obstruction, granuloma formation, and frequent relapse after stent removal. Compared to other treatment modalities, cryotherapy is significantly cost-effective and is a relatively simple procedure [11]. 

Overall, bronchoscopic cryotherapy is a simple procedure that is cost-effective, low-risk, and convenient compared to other treatment modalities and is a viable option for treating tracheal stenosis. Further studies, including randomized clinical trials, are required to validate the efficacy of cryotherapy in post-intubation tracheal stenosis. 

Limitations

A major limitation of our study is the inclusion of retrospective studies only. The small size of the study is also a limitation of our study. Most of the studies did not mention associated co-morbidities of the patient. There was a lack of uniform guidelines for the use of cryotherapy, and also an absence of comparison between other endoscopic and surgical procedures was noticed. Most of the studies did not mention the patient's follow-up after treatment. Further prospective studies are needed in this field to investigate and better understand these results.

## Conclusions

For patients with tracheal stenosis, multiple attempts to canalize are required. Whereas for poor surgical candidates, bronchoscopic Cryotherapy can be considered an alternative as it is easily performed with fewer side effects and excellent outcomes. But further studies and prospective trials are needed to validate the efficacy of cryotherapy for the patient with post-intubation tracheal stenosis.

## References

[1] Delaere P, De Leyn P (2019). Surgical anatomy of the trachea and techniques of resection and reconstruction. Shields' General Thoracic Surgery, 8th Edition.

[2] Beyoglu MA, Sahin MF, Turkkan S, Yazicioglu A, Yekeler E (2022). Complex post-intubation tracheal stenosis in COVID-19 patients. Indian J Surg.

[3] Stauffer JL, Olson DE, Petty TL (1981). Complications and consequences of endotracheal intubation and tracheotomy. A prospective study of 150 critically ill adult patients. Am J Med.

[4] Mathias DB, Wedley JR (1974). The effects of cuffed endotracheal tubes on the tracheal wall. Br J Anaesth.

[5] Sandu K (2021). Laryngotracheal complications in intubated COVID-19 patients. Clin Med Insights Case Rep.

[6] Yang KL (1995). Tracheal stenosis after a brief intubation. Anesth Analg.

[7] Sahin MF, Beyoglu MA, Yazicioglu A, Yekeler E (2022). Analysis of 40 patients who underwent tracheal resection due to benign complex tracheal stenosis. Asian J Surg.

[8] Bhora FY, Ayub A, Forleiter CM (2016). Treatment of benign tracheal stenosis using endoluminal spray cryotherapy. JAMA Otolaryngol Head Neck Surg.

[9] Fernando HC, Dekeratry D, Downie G (2011). Feasibility of spray cryotherapy and balloon dilation for non-malignant strictures of the airway. Eur J Cardiothorac Surg.

[10] Liu J, Zhang CP, Li Y, Dong S (2015). Post-intubation tracheal stenosis after management of complicated aortic dissection: a case series. J Cardiothorac Surg.

[11] Jung YR, Taek Jeong J, Kyu Lee M, Kim SH, Joong Yong S, Jeong Lee S, Lee WY (2016). Recurred post-intubation tracheal stenosis treated with bronchoscopic cryotherapy. Intern Med.

[12] Krimsky WS, Rodrigues MP, Malayaman N, Sarkar S (2010). Spray cryotherapy for the treatment of glottic and subglottic stenosis. Laryngoscope.

[13] Timman ST, Schoemaker C, Li WW (2018). Functional outcome after (laryngo)tracheal resection and reconstruction for acquired benign (laryngo)tracheal stenosis. Ann Cardiothorac Surg.

[14] Tayfun MA, Eren E, Başoğlu MS, Aslan H, Öztürkcan S, Katilmiş H (2013). Postintubation laryngotracheal stenosis: assessing the success of surgery. J Craniofac Surg.

[15] Chang E, Wu L, Masters J (2019). Iatrogenic subglottic tracheal stenosis after tracheostomy and endotracheal intubation: a cohort observational study of more severity in keloid phenotype. Acta Anaesthesiol Scand.

[16] Shin B, Kim K, Jeong BH, Eom JS, Song WJ, Kim H (2017). Clinical implications of differentiating between types of post-tracheostomy tracheal stenosis. J Thorac Dis.

[17] Rea F, Callegaro D, Loy M, Zuin A (2002). Benign tracheal and laryngotracheal stenosis: surgical treatment and results. Eur J Cardiothorac Surg.

[18] Sarper A, Ayten A, Eser I, Ozbudak O, Demircan A (2005). Tracheal stenosis aftertracheostomy or intubation: review with special regard to cause and management. Tex Heart Inst J.

[19] Kumar A, Asaf BB, Puri HV, Abdellateef A (2017). Resection and anastomosis for benign tracheal stenosis: single institution experience of 18 cases. Lung India.

[20] Greenwald BD, Dumot JA, Abrams JA (2010). Endoscopic spray cryotherapy for esophageal cancer: safety and efficacy. Gastrointest Endosc.

